# OMICS in Chronic Kidney Disease: Focus on Prognosis and Prediction

**DOI:** 10.3390/ijms23010336

**Published:** 2021-12-29

**Authors:** Michele Provenzano, Raffaele Serra, Carlo Garofalo, Ashour Michael, Giuseppina Crugliano, Yuri Battaglia, Nicola Ielapi, Umberto Marcello Bracale, Teresa Faga, Giulia Capitoli, Stefania Galimberti, Michele Andreucci

**Affiliations:** 1 Department of Medical and Surgical Sciences, University Magna Graecia of Catanzaro, Viale Europa, 88100 Catanzaro, Italy; rserra@unicz.it; 2 Interuniversity Center of Phlebolymphology (CIFL), Magna Graecia University, 88100 Catanzaro, Italy; 3 Department of Advanced Medical and Surgical Sciences, University of Campania Luigi Vanvitelli, 80138 Napoli, Italy; carlo.garofalo@unicampania.it; 4 Department of Health Sciences, Magna Graecia University, 88100 Catanzaro, Italy; ashourmichael@yahoo.com (A.M.); giusycrugliano2@gmail.com (G.C.); teresa_faga@yahoo.it (T.F.); 5 Division of Nephrology and Dialysis, St. Anna University-Hospital, 44124 Ferrara, Italy; battagliayuri@gmail.com; 6 Department of Public Health and Infectious Disease, Sapienza University of Rome, 00185 Roma, Italy; nicola.ielapi@uniroma1.it; 7 Vascular Surgery Unit, Department of Public Health, University Federico II of Naples, 80131 Napoli, Italy; umbertomarcello.bracale@unina.it; 8 Bicocca Bioinformatics Biostatistics and Bioimaging B4 Center, School of Medicine and Surgery, University of Milano-Bicocca, 20100 Milano, Italy; giulia.capitoli@unimib.it (G.C.); stefania.galimberti@unimib.it (S.G.)

**Keywords:** precision medicine, albuminuria, genomics, proteomics, metabolomics, SNP, chronic renal failure

## Abstract

Chronic kidney disease (CKD) patients are characterized by a high residual risk for cardiovascular (CV) events and CKD progression. This has prompted the implementation of new prognostic and predictive biomarkers with the aim of mitigating this risk. The ‘omics’ techniques, namely genomics, proteomics, metabolomics, and transcriptomics, are excellent candidates to provide a better understanding of pathophysiologic mechanisms of disease in CKD, to improve risk stratification of patients with respect to future cardiovascular events, and to identify CKD patients who are likely to respond to a treatment. Following such a strategy, a reliable risk of future events for a particular patient may be calculated and consequently the patient would also benefit from the best available treatment based on their risk profile. Moreover, a further step forward can be represented by the aggregation of multiple omics information by combining different techniques and/or different biological samples. This has already been shown to yield additional information by revealing with more accuracy the exact individual pathway of disease.

## 1. Introduction

Chronic kidney disease (CKD) is a common chronic disease which has been recognized as an independent predictor for cardiovascular (CV) fatal and non-fatal events, mortality from any cause, and kidney failure (also called end stage kidney disease, ESKD) [1,2]. The public health history of CKD has drastically changed in the past two decades, or even less, considering that in 2007 CKD was considered uncommon, untreatable, and hard to classify [3]. Since then, a large number of epidemiologic and clinical studies have been carried out and have revealed exactly the opposite. First of all, the prevalence of CKD in the general population is not trivial (i.e., higher than 10% as reported in the surveys in the US, Canada, Netherlands, China, and Australia), being even higher than that of type 2 diabetes [4,5]. More importantly, the prevalence of CKD is increasing worldwide, with the more recent Global Burden of Kidney Disease (2017) showing a global increase of 29.3% between 1990 and 2017 [6]. With respect to treatment, the past two decades have also seen a great implementation of intervention studies, with the completion of a number of clinical trials testing the efficacy and safety of novel drugs in CKD patients [7,8]. Major examples of novel developed drugs in CKD patients are the sodium-glucose cotransporter 2 inhibitors (SGLT2is) as well as the selective antagonists of endothelin-1 receptors (ERA) and the nonsteroidal mineralocorticoid receptor antagonists (MRA) [9,10,11]. These studies have shown a significant benefit of these novel pharmacological agents in protecting against CV and renal events in CKD patients [7]. Hence, the current state of play conceives CKD as a common and treatable disease with a solid system of classification based on albumin and eGFR. Nevertheless, although this seems like the end of the story, it is just the beginning. What complicates the management of CKD patients is the large intrinsic heterogeneity of CKD. In fact, CKD is triggered and perpetuated by a multitude of risk factors, from metabolic parameters to the presence of diabetes or immunologic factors, but the presence of each risk factor may elicit a different effect—and with variable severity—in different individuals. Owing to this evidence and to the increasing prevalence of CKD, the International Society of Nephrology (ISN) proposed to improve research on CKD by focusing on novel biomarkers that can help in predicting future events and in clarifying the pathways of disease [12]. To this end, one important opportunity is provided through the application of omics technologies. In fact, omics data derived from blood, urine and kidney biopsies may provide more information on molecular pathways of disease in CKD as well as help finding novel predictive and prognostic biomarkers useful to the management of CKD patients [13]. The aim of the present review is to summarize the available evidence of omics research in CKD and to discuss the future directions of such research in the context of personalized medicines [12,14,15].

## 2. Variability in Prognosis and Response to Treatments in CKD Patients

It has been well demonstrated that the presence of CKD exposes patients to an increased risk for all-cause mortality; CV events (such as myocardial infarction, stroke, heart failure, arrythmias, peripheral vascular disease); and CKD progression, namely the onset of the more advanced stage of CKD, known as ESKD, that often, due to the severe metabolic and hemodynamic complications involved, requires referral to renal replacement therapies (e.g., dialysis or kidney transplant). A series of four meta-analyses carried out by the CKD Prognosis Consortium, which synthetized data derived from the U.S general population, high-risk populations and patients with CKD, provided important evidence regarding this point [16,17,18]. In fact, low eGFR (<60 vs. ≥60 mL/min/1.73 m^2^) levels were associated with a two to threefold increased risk for all-cause and CV mortality independently of age, gender, proteinuria levels and other potential confounders. Similarly, the risk for ESKD increased exponentially moving from optimal eGFR to cut-offs of 45 mL/min or 15 mL/min [16,17,18]. The association between albuminuria and CV events, mortality and ESKD was linear with an increase of 1.5- to 2.5-fold higher risk at the cut-offs of mild and severe albuminuria [16] compared with normal albuminuria levels. Risk of ESKD was found to have increased by around 30 times when considering a cut-off of 1000 mg/g of albuminuria as compared with 5 mg/g [18]. Several other risk factors have been found to be associated with cardio-renal outcomes in CKD patients regardless of albuminuria and eGFR. Metabolic parameters, such as increased serum phosphorus or potassium levels, low hemoglobin levels, male gender, and increased systolic blood pressure, have been shown to forecast a negative prognosis in large studies enrolling CKD patients [19,20,21,22,23,24]. Despite several attempts to stratify future risks on the basis of baseline levels of all the aforementioned variables, the progression (and prognosis as well) of CKD is widely heterogeneous [12]. It has been shown that eGFR declines over time, which is a strong surrogate of CKD progression to ESKD, has a high degree of variability and is often unpredictable with the aforementioned traditional risk factors. In a pooled analysis of European CKD cohorts, eGFR decline ranged from 0.77 to 2.43 mL/min/year [25] on average, whereas in an Italian cohort of CKD patients referred to tertiary nephrology care, eGFR annual decline was −1.7 mL/min with an interquartile range of −4.6 to 0.8 mL/min [26]. A large variability in disease progression was also reported in randomized clinical trials despite the more stringent inclusion criteria of patients. In the Irbesartan Diabetic Nephropathy Trial (IDNT), which evaluated whether irbesartan or amlodipine slow the progression of CKD in patients with type 2 diabetes, the coefficient of variation for eGFR slope over time was 135% [27]. In the past two decades, a number of clinical trials have been completed with the aim to improve the treatment of CKD [9,10]. After the discovery of the protective effect of drugs interfering with the Renin-Angiotensin-Aldosterone System (i.e., RAAS inhibitors-RAASis) against the risk for CKD progression, a further step forward was reached more recently with the demonstration of the efficacy of novel treatments, such as SGLT2is (in DAPA-CKD, EMPA-REG, CREDENCE trials), MRA (FIDELIO-DKD trial) and ERA (SONAR trial) in patients with CKD [9,14]. Although such findings may be seen as a ‘landmark’ point for the treatment of CKD patients, it has been concomitantly demonstrated that a variability in response to these nephroprotective treatments exists and, even more importantly, it is non-negligible [28]. RAASis are now considered the standard of care for the management of proteinuria and non-proteinuric CKD, being prescribed in over 70% patients followed by nephrologists [29]. Despite their widespread diffusion, it has been demonstrated that about 30 to 40% of patients do not respond to RAASis [30]. Hence, response to antialbuminuric treatments varies between individuals, and is reproducible if the same drug (or a drug of the same class) is re-started in the same individual. Even more importantly, the variability in response is still present when a drug is up titrated to an optimal dose or when a drug is changed with another drug of the same family [31]. In addition to RAASis, the variability in response is a phenomenon that has also been reported for other nephroprotective drugs. Heerspink and colleagues showed that Asian patients, when compared with North Americans, responded better, in terms of albuminuria reduction over time, to the ERA atrasentan [32]. This difference has been attributed to a lesser likelihood of sodium retention after atrasentan initiation in Asian patients. Similarly, a variability in albuminuria reduction has also been shown in response to the SGLT2i dapagliflozin [32]. The ‘net’ result of this non-random variation of albuminuria is that a large proportion of CKD patients remain with persistent albuminuria and, therefore, at increased residual cardiovascular and renal risk [33]. The development and true validation of novel biomarkers is a crucial point in nephrology research, since this may help to improve both prognosis and prediction (of response to treatments) in individual CKD patients.

## 3. Omics: New Frontiers of Research

Biomarker discovery has been greatly implemented with the introduction of ‘omics’ techniques. Samples derived from kidney biopsies, urine and blood can be used to generate proteomic, genomic, metabolomic and transcriptomics biomarkers that may be more strongly and accurately linked to the pathophysiologic mechanisms of disease than traditional markers such as eGFR [34,35,36,37].

Proteomics has garnered a great interest since it may be well assessed using urine as the study sample. This approach has multiple advantages. In fact, urine samples are easy to collect and are almost routinely requested by clinicians for monitoring the response to treatment and for stratifying risk of future outcomes in patients with CKD [38,39]. Moreover, urine collection is non-invasive and with both first morning void collection and, in particular, with 24 h collection, a huge volume can be provided for proteomic analysis when compared with other biological fluids. Urine contains a large variety of proteins (there are about 1.8 million human proteoforms) which belong to various tissues and to the urogenital tract [40]. These proteins can be detected in urine as peptides, i.e., short amino acid sequences, or as more complex polypeptide molecules. Proteomics encompasses the analysis of peptides derived from enzymatic digestion or the entire molecules. Another approach is represented by the peptidomics that consists in characterizing the full pattern of peptides present in a specific sample, without previous enzymatic digestion [41]. Techniques that allow the characterization of structure and concentration of unknown molecules are mass spectrometry (MS) combined with ultra-performance liquid chromatography (UPLC) and capillary electrophoresis (CE). Since MS recognizes ionized molecules, a molecule ionization step is required prior to MS, and this can be performed by matrix-assisted laser desorption ionization (MALDI) or electrospray ionization (ESI). Genomics profiling involves the analyses of whole genome sequencing or whole exome sequencing (only the protein-coding region) using high throughput next generation sequencing (NGS) [42]. It is a rapid, economical approach that can be used to elucidate the actual cause of many genetically linked kidney diseases irrespective of the stages of disease, which is in contrast to renal biopsies, for example, that have often failed to identify a patient at the early or late stages of disease progression [43,44]. The specimen for genomic analyses can be collected from a tissue or biospecimen, such as serum or urine [45]. The United States Department of Food and Drug Administration (http://www.fda.gov/cber/guidelines.htm, last accessed: 14 October 2021) defined genomic markers as the quantifiable expression of gene function or gene regulation, which may consist of the expression of a single gene or a combined gene panel. For each case, an ideal genomic marker for CKD should correlate with the functional and structural changes in the kidney cells. Genomic profiling has unprecedented success in identifying the monogenic causes of CKD, with approximately 500 genes identified so far with the majority of these in the pediatric population [46]. In the adult population, inheritable kidney disease was also reported to attribute up to ~37% of all adult cases [47,48]. Furthermore, in a large genome-wide association study involving ~1 million people, there were 264 loci that were identified to be associated with eGFR and individual kidney function, which could be potential targets to characterize kidney disease progression and treatment allocation for these patients [49]. Metabolomic profiling enables a non-targeted quantitative measurement of a broad range of small molecules which are products of cellular metabolism (e.g., adenosine triphosphate, biogenic amine neurotransmitters, glucose, lipid signaling molecules) from the tissue or a bio-specimen (e.g., serum, urine, saliva) from a living organism [50,51,52]. It has been advantageous over genomic or proteomic analysis, since it is able to precisely reflect and provide a ‘snapshot’ of the current status in the cell function of an organism [53]. Moreover, metabolomic expression is an indicator of cellular changes or gene–environment interactions that are reflected in phenotypic changes and are possibly most easily amenable via drug treatment compared with modifying the genome or proteome [54,55]. With regards to analytical approaches for metabolomic analyses, high field nuclear magnetic resonance (NMR) and mass spectrometry (MS) coupled with capillary electrophoresis (CE-MS), liquid chromatography MS (LC-MS), or gas chromatography (GC-MS) are commonly used for identification, extraction, or quantification of metabolites [56]. However, it is important to note that the reproducibility of metabolomic analyses still remains a challenge, since it is often non-reproducible, due to variations in patient demographics, biospecimens used, and the analytical and computational approach [57,58,59]. Transcriptomics refers to the study of ribonucleic acid (RNA) in a population of cells or individuals and is used to improve knowledge around the activation of genes in different types of cells and pathologic conditions [60]. The transcriptome includes all the RNA transcripts present in a predefined sample, from messenger RNA (mRNA) to other RNA subtypes which do not code for proteins (non-coding RNA, ncRNA) but have well defined regulatory functions including transfer RNA (tRNA) and microRNA (miRNA). The two main techniques currently used for transcriptomics analyses are DNA microarrays and RNA sequencing [61]. Microarrays are glass layers where small DNA sequences (oligonucleotides) are arrayed. After sampling from control and experimental subjects, RNA is converted into cDNA and marked with fluorophores of different colors. The cDNA fragments are then hybridized with the oligonucleotides and the intensity and colors of the resulting signal allow us to understand where RNA expression is higher. RNA sequencing is an NGS technique which has the advantage of using small amounts of RNA and consists of RNA purification, creation of cDNA fragments (library) via reverse transcription and library sequencing. Another omics approach that may be applied is the so-called ‘microbiomics’. This is defined as the study of all microorganisms of a given community (i.e., the gut or the mouth in humans), as a whole. Microbiomics is usually performed by means of DNA extraction from a target sample, DNA amplification and sequencing. Next, DNA sequences are matched against sequence databases to achieve identification of the organism present. Several technologies allow the study of which genes these organisms express and the metabolic activities in progress [62]. Omics analyses are being embraced with the development of novel statistical methodologies for data analysis in general, since one of the characteristics of omics is the enormous quantity of information to process [63]. In the presence of a multitude of data and a small sample of subjects, finding significant associations between a biomarker (or a set of multiple parameters) with a prognostic or predictive endpoints may be hard. To this end, there are several data-driven methods which help to select the significant variables. Given a pre-defined list of possible variables, these methods select a final set of parameters which may be helpful in answering research questions. These include the penalized regression methods, such as least absolute shrinkage and selection operator (LASSO), which introduces restrictions to regression coefficients and thus is able to find an ideal subset of variables that predict an outcome or drug response [64] or the dimension reduction methods, such as principal component analysis (PCA) [65]. PCA is a statistical tool which allows the reduction of the dimensionality of data, while retaining all the relevant information of the variables included in the model. Principal component analysis is particularly helpful when one of the intended uses of a statistical model is the exportability from one sample to another, and in these cases the use of a low number of variables is preferred. Another sophisticated method that is increasingly used in clinical studies of chronic diseases is machine learning [66]. This encompasses a series of learning techniques (e.g., Support Vector Machine, k-nearest neighbor, neural networks, decision trees) that are able to learn from what happens in available observations and to detect a risk factor, a cause of disease or an endpoint in a new set of observations. This has attracted attention particularly for studies aimed at improving the classification and prognosis of chronic diseases.

## 4. Prognostic Omics in CKD

Several studies based on omics analysis have been carried out with etiologic or prognostic aims namely for improving risk stratification of CKD patients on future cardiovascular and renal events as well as to reach a better knowledge of pathophysiological mechanisms of disease. The general purpose of prognostic research in CKD is to find a biomarker that should be able to improve the prediction of an event beside and beyond the clinical intuition of clinicians and the already available laboratory and clinical parameters. In CKD patients, it has already been demonstrated that variables such as age, gender, eGFR and albuminuria (or proteinuria) levels allow a good prediction of renal and cardiovascular endpoints [67,68]. From their side, nephrologists have clinical tools that they use to evaluate the clinical status of CKD patients, such as the strict control of blood pressure, dietary intake, physical activity as well as adherence to pharmacological treatments [69,70]. However, the use of omics has been shown to achieve additional improvements in cardiorenal risk prediction. The first CE-MS analyses of the urinary proteome demonstrated that a panel of relatively few proteins (from 20 up to 50) allows discrimination between patients with different etiologies of renal diseases, such as IgA nephropathy, membranous nephropathy, focal and segmental glomerulosclerosis (FSGS), and diabetic kidney disease [71,72]. In the vast majority of cases, these proteins are represented by small fragments that are likely the products of proteolytic fragmentation of larger proteins [73]. Another CE-MS analysis of urinary proteomes conducted in 39 healthy subjects and 112 patients with type 2 diabetes detected polypeptides that are able to identify patients with type 2 diabetes and, even more importantly, to discriminate between patients with different degrees of proteinuria (from low-moderate to severe) [74]. A proteomic study, which involved only CKD patients, without the control group of healthy subjects, highlighted that urine peptide classifiers can identify with excellent sensitivity and sensibility the specific cause of CKD, thus differentiating between diabetic kidney disease from glomerulonephritis or nephrosclerosis [75]. The methodology of this study was rigorous since the authors identified a combination of multiple peptides, the so-called classifiers, for each CKD diagnosis and evaluated a huge number (1180) of urine samples. These discoveries, taken together, have important clinical and prognostic implications, since it has been well demonstrated that, among CKD patients, those with diabetic kidney disease and polycystic kidney disease have a higher risk of CKD progression and CV events compared with other glomerulonephritis or hypertensive nephropathies, in addition to an increased CV risk as proteinuria becomes more severe [38,76]. More recently, a panel of 273 peptides (thus called CKD273) has been developed by analysing the urinary proteome of both healthy subjects and patients with CKD from different etiologies [77]. This urinary marker was able to detect the presence of CKD irrespective for the etiology, and higher scores of CKD273 were cross-sectionally associated with a more severe individual risk profile as testified by the lower eGFR, lower haemoglobin, higher blood urea levels and increased urine protein excretion [78]. More intriguingly, CKD273 has been shown to predict future events in CKD patients. In fact, CKD273 performed well in predicting the eGFR decline over time and the onset of ESKD regardless of proteinuria and eGFR levels [78,79]. This suggests that CKD273 provided additional prognostic information when added to the two principal kidney measures, namely proteinuria and eGFR. Other than risk for CKD progression, CKD273 has also been shown to forecast CV events. In a recent time-to-event analysis, Verbeke and colleagues found that CKD273 was an independent predictor of fatal and non-fatal CV events including myocardial infarction, cerebrovascular events, cardiac and peripheral revascularization and heart failure in CKD stages I to III-b, namely the earlier stages of CKD, which need more prevention efforts compared with more advanced CKD stages where the fate of patients is almost unchangeable [80]. Proteomics analysis have also been performed in kidney tissues [73]. These studies showed a disparate expression of proteins in the renal cortex and renal medulla and detected the thymosin β4 as a marker of sclerosis in animal models of FSGS [81,82,83]. Nevertheless, the use of proteomics in CKD has been proposed to be an alternative to kidney biopsy in some clinical contexts, that would help the nephrologist to detect kidney disease early on, select the appropriate treatment and, hopefully, to monitor the treatment effect over time [84]. Many genetic variants have been associated with the presence of CKD and influence its prognosis. Several prognostic pieces of evidence can be extrapolated from these studies. In a genomic study involving over 1 million individuals from the Veteran Affairs population and the CKD Genetics Consortium, Hellwege and colleagues identified several variants that significantly explain eGFR variation [85]. Mutations in the PKD1gene, encoding polycystin 1, and the NOS3 gene, encoding nitric oxide synthase 3, have been significantly associated with variation in eGFR. The genome-wide association study (GWAS) also found single nucleotide polymorphisms (SNP) strictly associated with the severity of CKD. Several loci in fact explained the variation in serum uric acid levels (including TRIM46, INHBB, SFMBT1, TMEM171, VEGFA, BAZ1B) and urine albumin excretion (C9orf3 and variant rs334 of HBB encoding beta globin) [86,87]. This association acquires clinical significance when considering that both serum urates and urine proteins are two important CV risk factors in CKD patients [88]. Other SNPs, such as ZFHX3, PMF1-BGLAP, USP38, and TTBK1, have been associated with an increased risk for cerebrovascular accidents in CKD patients, thus helping to clarify the link between CKD and vascular disease [87]. The gene UMOD, encoding uromodulin, has also been associated with the severity of several diabetic and non-diabetic kidney disorders [89,90]. In particular, the variants rs77924615 and rs111285796 have been associated with an altered expression of UMOD in renal tubules of patients suffering from nephrotic syndrome, a clinical condition associated with increased CV risk given the high likelihood of developing thrombosis and ischemic vascular damage [91,92]. The variant rs955333 of CNKSR3 has been shown to influence sodium and water retention, and thus plasma volume expansion in patients with severe diabetic kidney disease [93]. CNKSR3 is involved in the regulation of the epithelial sodium channel (ENaC) located in the distal portion of the nephron, which mediates the sodium reabsorption in response to aldosterone. Variants of these genes may explain the increased sodium sensitivity present in patients with diabetic kidney disease that confers an increased CV risk to these patients [76]. In these patients, the variant Pro12Ala of the PPARG2 gene forecasted an increased risk for cerebrovascular events (defined as transient ischemic attack, cerebral ischemia, or cerebral haemorrhage) [94]. In CKD patients from different etiologies, the SNP rs495392 of the Klotho gene has been associated with a reduced risk for progression of atherosclerotic damage [95]. Klotho deficiency, as occurs during CKD progression, may contribute to endothelial dysfunction and arterial stiffness which, in turn, worsen the CV prognosis of CKD patients. In the Chronic Renal Insufficiency Cohort (CRIC), a cohort of CKD patients periodically followed for monitoring CKD progression, several genetic variants have been linked to the onset of future CV events [96]. Variants in chr9p21, COL4A1, ATP2B1 and HNF4A have been shown to predict coronary calcification and coronary artery disease over time in CKD patients. The products of these genes are involved in the regulation of blood pressure, vascular tone and calcium homeostasis and are thus strictly connected to CV complications in these patients. Some pharmacogenomic prognostic markers have also been reported thus far. The SNP Ser270Ala in SLC22A2 encoding the protein OCT2, a transporter of cationic drugs (such as metformin, metoprolol, cisplatin) located on the basolateral membrane of renal tubular cells, predicted the onset of side effects due the impaired urine excretion of these drugs [97,98]. Similarly, the variant Ala465Val of the SLC47A1 gene encoding MATE1 transporter has been associated with an impaired secretion of toxins and drugs with urine and thus with a higher frequency and severity of CKD [99]. Metabolomics is another field implemented in kidney diseases and has been considered as a useful tool since it simultaneously reflects the function of genes and proteins in biologic fluids [100]. The main aims of this field of research in nephrology are assessing whether alterations in the metabolome are associated with severity of CKD and whether they allow us to predict future events. Several previous studies revealed a significant role of plasma levels of kynurenine-to-tryptophan ratio, kynurenine and kynurenic acid in forecasting the onset of CKD, defined as an eGFR < 60 mL/min/1.73 m^2^ [101,102]. Nevertheless, in a subsequent longitudinal analysis of the CRIC cohort, none of these metabolites were associated with CKD progression [103]. Several other metabolites have been reported as good predictors for CV events. Using the LC-MS technique, it has been possible to isolate these metabolites, which are trimethylamine-N-oxide (TMAO), betaine and choline [104,105]. The plasma levels of TMAO were found to predict the incidence of stroke, CV acute events and mortality over time [106]. The pathway which links TMAO to CV risk has been confirmed in CKD patients and has been attributed to the fact that TMAO undergoes renal metabolism and thus its plasma concentration is strictly regulated by the kidney [106,107]. GC-MS has identified several urine metabolites of oxidative stress as being potentially involved in several patterns of kidney disease. Increased urine levels of fumarate, an intermediate of tricarboxylic acid cycle, and decreased urine levels of metabolites related to mitochondrial function have been found to be associated with an increased risk of developing diabetic kidney disease in mice [108]. Moreover, podocyte overexpression of NOX4, a major source of reactive oxygen species in the kidney, has been associated with diabetic kidney disease and a mechanism which directly involves the tissue levels of fumarate may be responsible for this pathway [109]. With respect to transcriptomics, several ncRNA have been found to be associated with the onset and progression of CKD [60]. A recent study carried out by Khurana and colleagues on 15 CKD patients and 10 healthy controls, showed that extraction of RNA from urinary exosomes led to the isolation of a number of ncRNAs that were differentially expressed in CKD and healthy subjects [110]. In particular, 9 miRNAs (miR-222-3p, miR-27a-3p, miR-27b-3p, miR-877-3p, miR-31-5p, miR-3687, let-7c-5p, miR-6769b-5p and miR-296-5p) were increased whereas 7 miRNAs (miR-133a, miR-133b, miR-15a-5p, miR-181a-5p, miR-34a-5p, miR-181c-5p and miR1-2) were decreased in CKD patients compared with healthy subjects. The authors suggested that some of these miRNAs could be evaluated as biomarkers of early diagnosis of CKD, in particular miR-181a which appeared the most reproducible and reliable miRNA in their analysis. Several miRNAs are involved in the dysregulation of extracellular matrix (ECM) deposition and epithelial-to-mesenchymal transition, two mechanisms which lead to renal fibrosis over time [111]. In diabetic nephropathy models, the increased expression of miR-192 determines an increased deposition of collagen via TGF-β, and this pattern is even amplified by miR-200. Clusters of miR-17-92 have been found to be associated with mesangial expansion, proteinuria and enlargement of renal glomeruli in the context of lupus nephritis [112]. In general, miRNAs have been found to be associated with dysregulation of podocytes, a mechanism shared by multiple causes of CKD, and determine a fast progression to ESKD and death [113,114]. Moreover, expression of miR-143 and miR-145 were found to be altered in an experimental model of atherosclerosis and CKD, thus suggesting a role of miRNAs in determining endothelial and vascular damage in CKD which is per se a condition associated with increased cardiovascular risk [115]. In the past decades, it has been demonstrated that gut microbiota have a relevant role in influencing human health [116]. In CKD patients, the microbiome is significantly altered in composition and diversity with an increase in bacteria which synthetize uremic toxins such as p-cresyl-sulphate and indoxyl sulphate. Meta-omics techniques have identified a number of peptides and proteins which are generated by fermentation in the large intestine and that their overexpression is associated with an increased risk for CKD progression and poor prognosis [117]. It has been postulated that some of these toxins such as p-cresyl-sulphate and indoxyl sulphate can be considered as biomarkers of CKD in the future [62].

## 5. Predictive Omics in CKD

**Figure 1:**
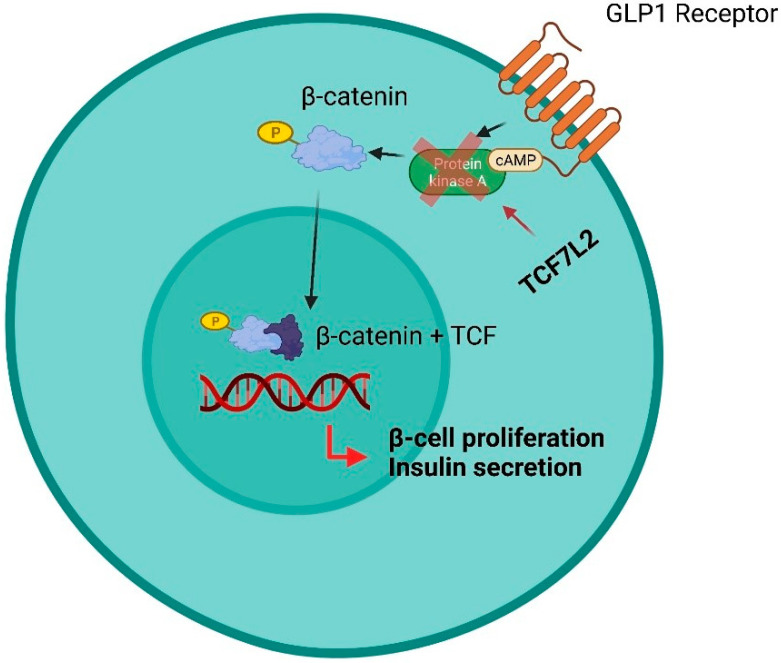
Influence of SNPs in the TCF7L2 gene in response to treatment with SU derivatives, GLP1-RA and DPP-4 inhibitors. In the pancreatic β-cell, GLP-1 phosphorylates β-catenin via cAMP-dependent protein kinase A (PKA). This avoids the degradation of the β-catenin that subsequently accumulates, enters the nucleus and forms the transcription factors β-catenin/TCF. This leads to activation of genes such as Isl-1 and Axin2. Overall, this pathway results in pro-insulin processing, β-cell protection from IL-1β and interferon-γ-mediated apoptosis, stimulation of β-cell proliferation and glucose/GLP-1-stimulated insulin secretion. Patients with TCF7L2 gene variants have impaired TCF7L2 expression in pancreatic β-cells resulting in reduced insulin secretion and impaired response to incretins (GLP1-RA, DDP-4 inhibitors). The abnormal response to GLP1-RA and DPP-4 inhibitors is likely to depend on the direct effect of TCF7L2 on PKA.

An ambitious role of omics in CKD is the detection of subjects who will likely respond to a specific treatment. This function can be similar to that of predictive biomarkers and confers on them the diction of predictive omics [118]. The evaluation and validation of predictive omics in nephrology is of great interest, given that a striking portion of CKD patients do not respond to nephroprotective treatments and novel interesting projects have been started but, to date, the available information is still incomplete. Patients with type 2 diabetes and CKD have been included in a cross-over randomized trial with two treatment periods with candesartan, an angiotensin II receptor blocker, or placebo, in random order [119]. As usually done in cross-over studies, at the end of each treatment period, which is followed by a wash-out that eliminates the effect of previous treatments before starting a new drug, laboratory or clinical measurements were performed. In this study, the treatment effect was associated with a change in a urine pattern of damage consisting of 113 polypeptides. Treatments with candesartan modified 11 out of 113 polypeptides and the degree of changes was positively correlated with the degree of change in albuminuria across treatments, thus giving even more emphasis to the results. These findings are important in the context of CV risk reduction, since predicting the reduction of albuminuria in short treatment periods is considered as a good surrogate marker of CV protection in the long term [38,39]. After demonstrating a good prognostic performance in CKD patients, the CKD273 classifier has been evaluated as a predictive omics in randomized trials scenarios. CKD273 has been proposed as a useful tool for biomarker-based enrichment studies, namely clinical trials, in which patients are considered eligible, and thus included, based on the baseline levels of a prognostic marker [12]. The rationale of this approach consists of including in intervention studies those patients who are at increased risk for developing future negative outcomes. In the ‘proteomic prediction and renin angiotensin aldosterone system inhibition prevention of early diabetic nephropathy in type 2 diabetic patients with normoalbuminuria’ trial, patients were enrolled based on increased risk of developing albuminuria according to the CKD273 score [120]. High-risk patients were then randomized to receive spironolactone or placebo and the development of mild albuminuria was the primary study outcome. Although spironolactone did not confer a significant reduction of albuminuria risk, likely because of discrepancies between the sample size calculated and the number of patients subsequently allocated in the high-risk group, nonetheless the high-risk group, identified with the CKD273, was at significantly higher risk of developing albuminuria than the low-risk group (*p* < 0.001). In fact, other studies revealed a significant change in CKD273, toward the values observed in healthy subjects, after treatment with RAASis or MRA [121,122]. Hence, this study is an original example of how omics can be used in the early phase of disease, and in fact PRIORITY trial patients, who were not yet albuminuric, were treated with albuminuria-lowering drugs. Another interesting result derives from a post-hoc analysis of the Efficacy, Safety & Modification of Albuminuria in type 2 Diabetes Subjects with Renal Disease with the LINAgliptin (MARLINA-T2D) trial [123]. This trial originally demonstrated the efficacy in terms of glycemic control of linagliptin, a dipeptidyl peptidase-4 inhibitor, when added to standard treatment in patients with diabetic kidney disease. Interestingly, patients with high-risk score according to CKD273 and who started linagliptin were those who benefitted more from this treatment in terms of loss of renal function and, more importantly, this benefit was not captured by eGFR alone. Many attempts to assess the predictive role of proteomics have been made with respect to IgA nephropathy. Several studies suggest a role of urine kininogen and urine proteomic classifiers in predicting the response to RAASis treatment, which needs further confirmation from larger intervention studies [75,124]. Genetic variants in several crucial loci have also shown predictive, other than prognostic, ability. An insertion (I) or deletion (D) polymorphism of the angiotensin-converting enzyme gene influences the renal and systemic function of RAAS. Some analyses showed that these polymorphisms may influence the response to RAASis and, consequently, may affect the cardiorenal prognosis in CKD patients [125,126]. In particular, the treatment effect of ramipril and losartan, in the REIN and RENAAL trials, respectively, was greatest in patients with DD phenotype compared with ID or II genotypes. The relative risk reductions were large, being 61.3% in the REIN study and 41.3% in the RENAAL study. It is remarkable that such analyses have been carried out specifically in patients with CKD. Single nucleotide polymorphisms in the gene regulating cytochrome P450 function, and hepatic metabolism (SLCO1B1, ABCB1, ABCC2, ABCG2 and ABCB11) modifies the response to statins, which are frequently used in CKD patients to reduce CV risk [127,128]. Many other SNP variants have been found to be associated with variable responses to other nephroprotective treatments. Genetic variants in the UGT1A9 gene encoding the uridine 5’-diphospho-glucuronosyltransferase (UGT) enzyme influence the response to canagliflozin and other SGLT2is. In fact, patients who are carriers of the variants UGT1A9*3 and UGT2B4*2 have a higher AUC of canagliflozin and thus a better drug availability [129,130,131]. Variations in the TCF7L2 gene have reduced insulin secretion from pancreatic β-cells and thus have an impaired response to incretins (dipeptidyl peptidase-4 inhibitors—DPP-4—and glucagon like peptide 1 receptor agonists—GLP1-RA), which have been shown to confer CV and renal protection in patients with diabetic kidney disease (Figure 1) [132].

Furthermore, metabolomics biomarkers have been proposed to be candidates as good predictors for response to treatment. In experimental models, niacinamide gained particular interest since its mediator, the PPARγ-coactivator-1α (PGC1α), is quantifiable in renal tissue and urine as an omics biomarker. The intraperitoneal infusion of niacinamide increased NAD levels in the kidney, a coenzyme critical for activating energetic metabolism and which may offer protection against kidney failure and CV risk [133]. The involvement of miRNAs in many crucial mechanisms of damage in CKD has sparked great interest in developing drugs that block specific miRNAs or their function. The available tools to inhibit miRNAs are sponges, which absorb miRNAs reducing their activity, or oligonucleotides, which are more stable in vivo and thus also more used [134]. This latter strategy is very intriguing since, after systemic circulation, the oligonucleotides accumulate in the kidney and so exert a prolonged local action. The inhibition of miR-21 has been tested as treatment for reducing fibrosis in animal models and has been shown to reduce the severity of renal dysfunction, albuminuria and inflammation in a genetic model of Alport syndrome [135]. In a diabetes mouse model, the inhibition of miR-192 reduced the development of diabetic kidney damage, but the effect in humans is still controversial [136,137]. Replacement treatment with miR-145 has been shown to stabilize atherosclerotic plaques in mice [60]. Finally, but very importantly, the treatment-induced modification of gut-derived toxins has been considered as a promising strategy to reduce risk for CKD progression. A number of intervention studies have been carried out to evaluate whether the use of probiotics, namely microorganisms beneficial for the microbiome, may improve CKD progression and whether this effect can be associated with a change in biomarker levels [70,117]. Probiotics administered are mainly represented by Bifidobacterium, Lactobacillus, and Streptococci species. It has been shown that treatment with probiotics specifically in CKD patients in stage G3a determined a significant reduction of urinary levels of 3-methyl-indole and indican, two markers of intestinal dysbiosis. Further promising results derive from the benefit from treatment with AST-120, an oral spherical activated carbon, which absorbs small-molecule uremic toxins, leading to ESKD risk reduction. However, more studies are needed to clarify the individual response to probiotics in larger CKD populations [138]. Table 1 reports a summary of prognostic and predictive OMICs in CKD patients.

## 6. Omics and Personalized Medicine in Nephrology: Future Perspectives and Conclusions

Prediction of cardiovascular outcomes in CKD patients needs to be improved. A number of initiatives have been implemented in the general population with the aim of improving individual awareness of CV damage and reinforcing prevention strategies. The Joint British Societies launched a self-assessed risk score where patients are allowed to personally select those risk factors that deserve to be improved [139]. In the nephrology field, data are sparse, and risk scores in patients without CKD cannot be directly applied to those with CKD, as shown for the Framingham risk score [140]. For this reason, the International Society of Nephrology pushed the implementation of novel risk scores which are more appropriate to predict CV events in CKD patients through (1) the construction of large and multicentric databases, (2) the amelioration of statistical methodology while building a risk score with the assessment of external validation, calibration, GIF and discrimination and (3) the introduction and development of novel biomarkers that are able to add useful information for CV risk prediction [14]. The omics strategies can be involved in all these steps. However, a great effort is still necessary in this direction [141,142]. The main limitations of omics studies in CKD are the small sample size and the short follow-up in available studies. In fact, the vast majority of evidence available thus far is focused on the demonstration of change in biomarker levels (i.e., urine albumin excretion) after some months from the baseline study visit. In nephrology research, the major endpoints, such as ESKD or all-cause mortality, may need years to occur, especially if enrolled populations include young patients and those with a mild reduction of kidney function. Further studies with longer follow-up and a higher number of patients are needed. For all types of omics signatures, namely proteomics, genomics or metabolomics, future studies should be oriented at demonstrating prognostic and predictive abilities specifically in CKD patients. A clear example of the strict relationship between predictive and prognostic biomarkers is the importance of CKD273 that may allow a better risk stratification of patients and the identification of CKD patients who will respond better to a specific treatment.

As we mentioned in the previous sections, a lot of evidence has been provided thus far but, in most cases, they have been assessed in settings different from CKD patients. This is a key point since CKD patients differ from the general population and other high-risk patients (type 2 diabetes or hypertensive patients) for baseline characteristics, risk factors, and long-term prognosis [143,144,145]. In fact, CKD patients, despite an advanced mean age, are more prone to develop ESKD than mortality as outcome and have a high-risk CV profile (frequency of diabetes, smoking habit, previous CV disease) at baseline [146,147,148]. Moreover, the omics approach should be able to provide additional information not only on prediction and prognosis, but also on the pathogenetic mechanisms of CKD. An interesting strategy that would help to do this is to aggregate data derived from renal tissue and/or urine samples into networks that can provide more information of specific pathways of kidney disease [12]. For example, the integration of genetic data with histological, molecular data and clinical variables (the so-called multi-layered datasets) may help to better understand the mechanisms of kidney disease and to select the best therapeutic option for each patient. Analytic tools that have been proposed to build such datasets are: RNA sequencing of compartment-specific tissue (e.g., glomeruli or tubules in the kidney) to generate transcriptomic data; implementation of digital imaging of kidney biopsies that may generate high quality images and apply computer-aided techniques such as machine-learning [37]; multiple omics assessments from urine samples. An integrated omics approach has already been used in research. Following such an approach, one study demonstrated that genetic expression of EGF, encoding the protein epidermal growth factor (EGF) in kidney tissue (derived from biopsy) correlated significantly with kidney measures such as eGFR reduction or kidney failure, and also with urine levels of EGF [149]. Moreover, in a cohort of 157 European patients with CKD from multiple etiologies, the investigators integrated the eGFR-associated genetic loci information with transcriptional data [150]. Through such a study, it was possible to identify activated and suppressed inflammatory, metabolic and immune processes across different causes of CKD (i.e., diabetic CKD, several forms of glomerulonephritis). The patterns discovered involved the NF-E2-related factor 2 and JAK-STAT pathway. Interestingly, these data have been confirmed in multiple cohorts which reported that loss of EGF expression in the kidney was associated with a high-risk of CKD progression, thus providing important prognostic information. The integrated multi-layered approach should be also important to assess whether omics analysis can completely substitute for renal biopsy or also whether renal biopsy remains an important and unique diagnostic and prognostic tool for CKD patient management; this is still a matter of debate [151]. In one study, the information derived from kidney biopsies have been merged with genome-wide transcriptional analysis [152]. This study involved 95 human biopsies and demonstrated that there was a differential gene expression (with respect to CPT1A, CPT2, ACOX1-2) between kidney samples with and without fibrosis. Such a discovery is relevant as the degree of renal fibrosis is one of the main parameters used to guide treatment decision among nephrologists.

Another important aspect of omics research is related to the clinical trials design for personalized medicine. There are several clinical trial designs which are currently used in evaluating the efficacy and safety of nephroprotective treatments at an individual level, i.e., by focusing on selecting the ‘right drug for the right patient’ [12]. Three main examples of these studies are represented by: (1) The biomarker-based enrichment in which patients are selected on the basis of the presence/absence of a biomarker. (2) The adaptive enrichment studies in which all patients undergo a phase of experimental treatment (e.g., enrichment period) and only patients who respond to the study treatment will next enter the randomization to treatment or placebo. In such studies, treatment response could be based on the changing of specific blood or urine markers. (3) The cross-over designs in which patients undergo two or more treatment phases (with different drugs) separated by wash-out periods and the response to treatment in each phase is assessed by measuring the change of marker levels from the start to the end of treatment. The omics markers can be implemented in all these study designs by giving information as to which patients are likely to respond to a treatment.

## 7. Conclusions

In conclusion, omics research in CKD patients has been started and has provided much important information. Significant steps forward have indeed been made with respect to both prognosis and prediction of future CV and renal events in CKD. However, future studies may be oriented toward the correct definition of the role of omics specifically in CKD patients, the implementation of multi-omics integrated approaches and, hopefully, the integration of omics in the novel designs of randomized clinical trials.

## Figures and Tables

**Table 1:** Prognostic and predictive OMICs in chronic kidney disease.

Name	Types	Source	Role	Outcome	Disease	Note/Comments
Human studies						
CDK273 [78,79]	Peptide	Urine	Prognostic	ESKD onset or eGFR decline; CV events	CKD	High CKD273 score was associated with an increased individual risk for CKD progression.
PKD1 & NOS3 [85]	Gene	Single nucleotide polymorphisms (SNPs)	Prognostic	Decrease in renal function (eGFR)	Renal function	Mutations of PKD1, encoding polycystin-1 and NOS3, involving nitric oxide production, have been associated with reduced renal function.
TRIM46, INHBB, SFMBT1, TMEM171, VEGFA, BAZ1B [86,87]	Gene	Single nucleotide polymorphisms (SNPs)	Prognostic	CV events	CKD	SNPs in these genes influence serum uric acid levels and this association partially explains the increased CV risk in CKD.
C9orf3 and variant rs334 of HBB encoding beta-globin [86,87]	Gene	Single nucleotide polymorphisms (SNPs)	Prognostic	CV events	CKD	These genetic variants influence urine albumin excretion and mediate the association between CKD and CV events.
ZFHX3, PMF1-BGLAP, USP38, and TTBK1 [87]	Gene	Single nucleotide polymorphisms (SNPs)	Prognostic	Cerebrovascular accidents	CKD	SNPs in these genes influence serum uric acid levels and this association partially explains the increased risk for cerebrovascular accidents in CKD patients.
UMOD [91,92]	Gene	Uromodulin	Prognostic	Disorders in diabetic nephropathy	Diabetic nephropathy	The variants rs77924615 and rs111285796 were found to predict the risk of nephrotic syndrome.
PPARG2 [94]	Gene	Single nucleotide polymorphisms (SNPs)	Prognostic	Cerebrovascular events	CKD patients	Variant Pro12Ala was able to predict cerebrovascular events in CKD patients.
Klotho [95]	Gene	Single nucleotide polymorphisms (SNPs)	Prognostic	Progression of endothelial dysfunction and arterial stiffness, CV events	CKD	Genetic variants of Klotho gene influence CV risk and progression of atherosclerosis in CKD patients.
Chr9p21, COL4A1, ATP2B1, and HNF4A [96]	Gene	Single nucleotide polymorphisms (SNPs)	Prognostic	CV events	CKD	These genes are involved in regulation of blood pressure, vascular tone, and calcium homeostasis and their variants predict coronary artery disease in CKD patients.
MATE1 [99]	Gene	Single nucleotide polymorphisms (SNPs)	Prognostic	Severity of CKD	CKD	MATE1 secretes drugs from cells into the lumen of proximal tubules. Several genetic polymorphisms such as the variant Ala465Val of SLC47A1 may affect the function of this transporter with impaired secretion of toxins and drugs, which reflect on the severity of CKD.
Trimethylamine-N-oxide (TMAO) [105]	Amine oxides	Serum	Prognostic	Stroke, CV acute events, and mortality over time	CKD	Trimethylamine-N-oxide (TMAO) plasma levels are strictly associated with the incidence of stroke, CV acute events, and mortality over time. TMAO levels increase with the progression of kidney damage. Therefore, this marker could perform even better in CKD patients.
NOX4 [109]	Gene	Single nucleotide polymorphisms (SNPs)	Prognostic	Severity of CKD	CKD	NOX4 expression increases fumarate levels, which are linked to glomerular dysfunction. Therefore, fumarate is a key link connecting metabolic pathways to diabetic nephropathy.
miR-222-3p, miR-27a-3p, miR-27b-3p, miR-877-3p, miR-31-5p, miR-3687, let-7c-5p, miR-6769b-5p miR-296-5p miR-133a, miR-133b, miR-15a-5p, miR-181a-5p, miR-34a-5p, miR-181c-5p miR1-2 [111]	miRNAs	Non-coding RNA fragments	Prognostic	Severity of CKD	CKD	These miRNAs are differentially expressed in CKD patients. miRNAs associated with CKD impair the degree of fibrosis, ECM deposition and proteinuria and accelerate CKD progression.
p-cresyl-sulphate indoxyl sulphate [117]	Protein-bound uremic toxins	Microbiomics	Prognostic	Severity of CV damage	CKD	Overexpression of uremic toxins accelerate CKD progression.
CKD273 [120,121,122,123]	Peptide	Urine	Predictive	Response to RAASi/DPP-4	Diabetic nephropathy	CKD273 panel is not only a prognostic but also a predictive tool. There is a close relationship between a high CKD273 score and response to RAASi or Linagliptin therapy. In high-risk patients undergoing therapy with the latter, the CKD273 score had a significant decrease compared with healthy subjects.
Urine kininogen [75,124]	Peptide	Urine	Predictive	Response to RAASi	CKD	Urine kininogen could predict the response to therapy with RAASi. However, further studies are needed.
Angiotensin-converting enzyme gene polymorphisms [125,126]	Gene	Single nucleotide polymorphisms (SNPs)	Predictive	Response to RAASi	CKD	Polymorphisms (insertion or deletion) for the gene encoding the angiotensin-converting enzyme may predict the response to RAASi. In one study, the D/D variant, followed by the I/D variant, resulted in a greater reduction in proteinuria, and better renal function over time. In contrast, the I/I variant predicted poor response and less benefit from RAASi therapy.
SLCO1B1, ABCB1, ABCC2, ABCG2 and ABCB11 [127,128]	Gene	Single nucleotide polymorphisms (SNPs)	Predictive	Response to statins and consequent increased CV risk	CKD	Several polymorphisms (SLCO1B1, ABCB1, ABCC2, ABCG2, and ABCB11) for the gene encoding cytochrome P450 could affect the response to statins, which play a central role in reducing CV risk among CKD patients.
UGT1A9 [129,130,131]	Gene	Single nucleotide polymorphisms (SNPs)	Predictive	Pharmacokinetics of SGLT2i	CKD and diabetes	UGT1A9 gene translates for an enzyme involved in the pharmacokinetics of SGLT2i. Carriers of the variants UGT1A9*3 and UGT2B4*2 have higher plasma levels of drugs, which are associated with greater benefits.
TCF7L2 [132]	Gene	Single nucleotide polymorphisms (SNPs)	Predictive	Pancreatic response to incretins	CKD	Some variants of the TCF7L2 gene cause a lower pancreatic response to incretins. Therefore, patients carrying these variants are expected to benefit less from therapy with GLP-1 agonists or DDP-4 inhibitors.
miR-192 [137]	miRNAs	Non-coding RNA fragments	Predictive	Onset of CKD	CKD	Inhibition of miR-192 reduces the renal complications of diabetes.
3-methyl-indole indicant [70]	Protein-bound uremic toxins	Microbiomics	Predictive	Severity of inflammation	CKD	Reduction of urinary levels of both markers after treatment is associated with reduction in inflammatory patterns in CKD patients.
Animal studies						
Thymosin β4 [81,82]	Protein	Renal parenchyma	Prognostic	Sclerosis progression	Segmental glomerulosclerosis (FSGS)	Thymosin β4 was associated with sclerosis progression in animal models of FSGS.
miR-143 miR-145 [115]	miRNAs	Non-coding RNA fragments	Prognostic	Severity of CV damage	CKD	They are associated with higher severity and less stability of atherosclerotic plaque in CKD.
miR-21 [135]	miRNAs	Non-coding RNA fragments	Predictive	Severity of CKD	CKD	Inhibition of miR-21 reduces renal fibrosis in Alport nephropathy.
miR-145 [60]	miRNAs	Non-coding RNA fragments	Predictive	Severity of CV damage	CKD	Inhibition of miR-145 allows stabilization of the atherosclerotic plaque and the onset of CV events.

## Data Availability

Not applicable.
